# Static and Flexural Fatigue Behavior of GFRP Pultruded Rebars

**DOI:** 10.3390/ma14020297

**Published:** 2021-01-08

**Authors:** Michał Barcikowski, Grzegorz Lesiuk, Karol Czechowski, Szymon Duda

**Affiliations:** Faculty of Mechanical Engineering, Wroclaw University of Science and Technology, PL 50-370 Wroclaw, Poland; Michal.Barcikowski@pwr.edu.pl (M.B.); 222000@student.pwr.edu.pl (K.C.); Szymon.Duda@pwr.edu.pl (S.D.)

**Keywords:** GFRP, rebars, composite, compression test, fatigue, flexural strength

## Abstract

This paper presents the experimental results of composite rebars based on GFRP manufactured by a pultrusion system. The bending and radial compression strength of rods was determined. The elastic modulus of GFRP rebars is significantly lower than for steel rebars, while the static flexural properties are higher. The microstructure of the selected rebars was studied and discussed in light of the obtained results—failure processes such as the delamination and fibers fracture can be observed. The bending fatigue test was performed under a constant load amplitude sinusoidal waveform. All rebars were subjected to fatigue tests under the *R* = 0.1 condition. As a result, the S-N curve was obtained, and basic fatigue characteristics were determined. The fatigue mechanism of bar failure under bending was further analyzed using SEM microscopy. It is worth noting that the failure and fracture mechanism plays a crucial role as a material quality indicator in the manufacturing process. The main mechanism of failure under static and cyclic loading during the bending test is widely discussed in this paper. The results obtained from fatigue tests encourage further analysis. The diametral compression test reflects the weakest nature of the composite materials based on the interlaminar compressive strength. The proposed methodology allows us to invariantly describe the experimental transversal strength of the composite materials. Considering the expected durability of the structure, the failure mechanism is likely to significantly improve their fatigue behavior under the influence of cyclic bending. The reasonable direction of searching for reinforcements of composite structures should be the improvement of the bearing capacity of the outer layers. In comparison with steel rebars (fatigue tensile test), the obtained results for GFRP are comparable in the HCF regime. It is worth noting that in the near fatigue endurance regime (2–5 × 10^6^ cycles) both rebars exhibit similar behavior.

## 1. Introduction

Rapid growth in the civil engineering field has enforced the development of new technologies. Composite bars FRP (Fiber Reinforced Polymers) are used in the process of concrete structure reinforcement, and they are a good example of dynamically developed material in recent years. Węglowski [1] gives examples of the application of composite rebars in modern constructions, mostly as reinforcement in bridge decks, acoustic barriers, and underground reinforcement. Moreover, this reinforcement is widely used in constructions such as swimming pools, airports, parking lots, and soft-eye (cavity) walls in underground tunnels [2,3,4,5].

The composite rebars provide advantages including high specific strength; by applying various resins and fibers they may fit specific requirements. The composite rebars prices plummet due to the expansion of the manufacturing technologies, used more often in civil engineering. The expansion of composite rebars application significantly increased in the early 1990s. Initially, they were applied in Western Europe, the USA, Japan, and Canada [6]. The external constructions and projects based on this composite reinforcement showed profitable results in terms of many experiments and proved that composite rebars might be an alternative for steel bars, and a breakthrough should be expected in their applications [7]. In comparison with steel rods, FRP rods offer several advantages such as greater tensile strength at a lower strain, the unit mass of the composite rods oscillates from a/one-sixth to a/one-quarter of the steel bars [8]. These properties decrease transportation costs and favor the construction reinforcement process. Moreover, additional advantages of these rods are high corrosion resistance, and the possibility of application in the toughest conditions, because they are not exposed to the same chemical environment as steel rods. Due to these significant advantages in recent years, the demand for composite rebars increased all over the world. In the market there have appeared a lot of new companies dealing with the manufacturing of the composite rebars using various material components in a changed ratio in terms of diverse pultrude processes consequently, the different geometric, physical, and mechanical properties can be obtained. Moreover, the manufacturers developed various methods of refining reinforcement connection with concrete, which is a crucial aspect of applying them in concrete structures [9,10].

In the literature, extensive research has been conducted on concrete structures and their strengthening. Authors such as De Lorenzis et al. [11] and Szabó et al. [12] cover this aspect in their studies. De Lorenzis and Szabó investigated the near-surface mounted (NSM) fiber-reinforced polymer (FRP) reinforcement technique for reinforced concrete (RC). This relatively new method may be an alternative for externally bonded FRP reinforcement in terms of economical and strength aspects. Casalegno et al. [13] present a study on the retrofit of traditional masonry using pultruded GFRP. This work shows that composite rebars are a viable alternative for old structures offer the advantages of lightness and durability of FRP reinforcement in comparison with steel frame retrofits. One of the frequently investigated aspects considering composite rebars is the rehabilitation of old bridges mentioned by authors such as Majumdar et al. [14] and Weber et al. [15].

Despite many variant forms of FRP rebars offered by manufacturers, and many experiments conducted on structures reinforced with these elements, this kind of reinforcement on the civil market is called unconventional [16]. The major reason is there were no global standards and recommendations (similar to for the steel market) on how to design the construction reinforcement with composite rods. Recently, in 2017 the American Society for Testing and Materials (ASTM) issued the first standard with the designation D7957/D7957M-17 [17] describing the mechanical properties of composite rebars. In contrast to steel reinforcement, for composite rods there are not as many standards. This aspect forces engineers to conduct authorial tests for each structure separately. Due to the possibility of shaping the rebar geometry during the pultrusion process, many authors were encouraged to produce a variety of rebar shapes [18,19,20,21]. However, their research was focused on static tests, which often consisted of organic solution—without evaluating the long-term strength characterized by fatigue tests of the bars themselves as components of reinforced concrete (RC). It is worth noting, however, that in the RC, the problem of fatigue becomes much more complicated due to the problems of fatigue of RC components and adhesion between bars and concrete. Many works focus on static flexural behavior [22,23,24] and widely on cyclic loading [25,26,27,28,29] of complex structures (RC + GFPR rebars). However, in several parts, there is a lack of fatigue tests for “pure” composite rebars. Despite these impediments, the FRP rods in a promising and positive alternative for conventional steel reinforcement.

This paper aims to bridge this gap using GFRP rebars during the cyclic flexural test. The fatigue experiments allow to describe the S-N behavior for the investigated composite rebars manufactured by ANKRA LLC, Poland. The obtained results fill the gap in the literature and allow for the assessment of the decohesion fatigue mechanism for various stress levels. Also, they allow for a better understanding of the nature and field of application (also limitations) of GFRP in the reinforcement of structures. This assessment gives various fractographic views of microsection surfaces that proves the multifacetedness of fatigue processes in the composite materials.

The objective of the paper is formulated as a fatigue strength assessment of two types of reinforcement rebars. Static bending, radial compression, and flexural fatigue test are performed. These tests reflect the loading modes of actual reinforcing bars in concrete structures—flexion from the global deformation of the structural element, and transverse compression from local pressure transferred through the concrete. Additionally, the analysis of the failure and fracture mechanism of these rebars was investigated in terms of the base material used in the manufacturing process.

## 2. Materials and Methods

### 2.1. FRP Reinforcement

Composite rebars (FRP) are marked by fair mechanical properties such as great tensile strength, and physical properties such as lower density in comparison to steel reinforcement. They have instanced the deployment in the objects particularly exposed to aggressive environments, and such where the proper working enables electromagnetic indifference, including construction. To reach a high corrosion resistance and electromagnetic indifference, a suitable material should be used for manufacturing not only the longitudinal reinforcement (to carry the bending load) but also the reinforcement to carry the transverse forces [30]. The basic properties are presented in Table 1.

According to the ACI 440.1R standard [31], FRP reinforcement is characterized by over 4-times lower density compared to steel, which significantly influences reinforcement works and decreases transportation costs. The thermal expansion is taken into account and the steel is an isotropic material, whereas the behavior of FRP rods is dependent on the fiber direction. The type of fiber decides on longitudinal thermal expansion of reinforcement, and the transverse thermal expansion depends on the resin type [17].

Composite rebars show elastic-brittle tensile strength characteristics. In contrast to the classic steel reinforcement, composite rebars are characterized by greater tensile strength oscillating between 600 and 3700 MPa, and relatively low Young Modulus between 35 and 125 GPa, excluding carbon fiber reinforcement polymer (CFRP).

Table 2 presents selected typical mechanical properties in a parallel direction to fibers [17].

**Figure 1:**
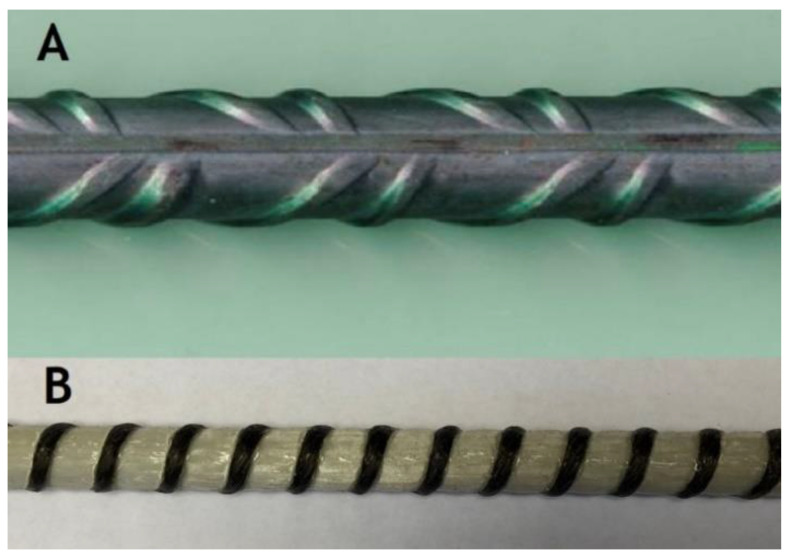
Concrete reinforcement–rebars subjected to mechanical testing; (**A**) B500SP steel, (**B**) GFRP rebar manufactured by Ankra company (Poland).

For the experimental campaign, steel rods were devoted, as well as composite rebars. The rods with a 10 mm diameter were used for experimental testing (steel and GFRP), as presented in Figure 1.

Composite rebars subjected to the experimental campaign are made from glass fibers in an epoxy resin matrix with the fibers content of 65–70%. Moreover, an over-wrap is applied over the plastic rods to increase the buckling and flexural strength [32,33]. The parameters of this rebar are shown in Table 3.

For each conducted experiment, the samples needed to be cut to the proper length. An accurate cut was required to obtain the accuracy of the results as best as possible. The specimens were cut for specific length using metallographic cutters METACUT 302 (METKON, Bursa, Turkey). It cuts with the manual feed of the grinding wheel.

Moreover, the structural research using a scanning electron microscope required appropriately prepared samples including mounting and burnishing using grinder-polisher FORCIPOL 2V (METKON, Bursa, Turkey). The objective of mounting was to stabilize the sample in the neutral material (ELECTROMIX resin) allowing the controlled machining.

**Figure 2:**
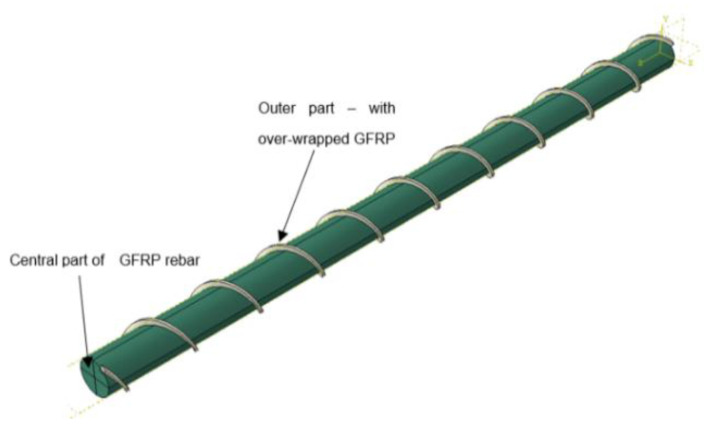
Locations of microscopic examination areas from the GFRP rebar.

The microstructure of the composite reinforcement was investigated before and after the fatigue tests. For microscopic analysis specimens were selected from outer and central parts of rebars, as is shown schematically in Figure 2. The longitudinal and transverse microsections were prepared to investigate the variations in microstructures.

**Figure 3:**
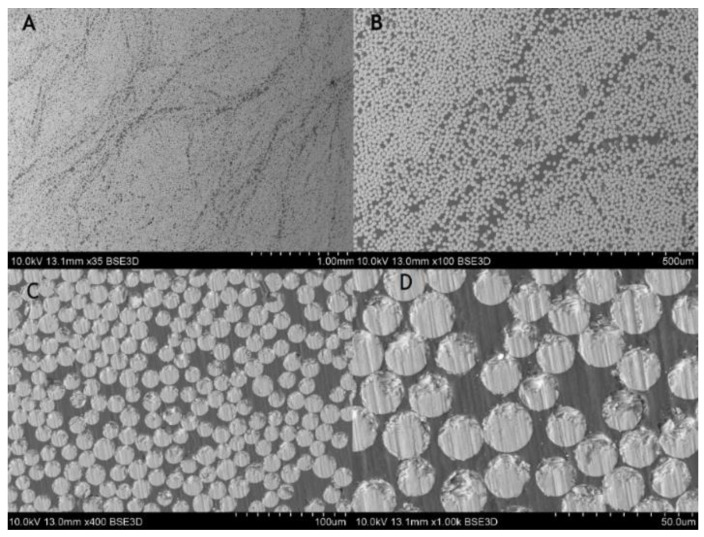
SEM images of composite rebar’s cross-section using (**A**) 35 times magnification, (**B**) 100 times magnification, (**C**) 400 times magnification, (**D**) 1000 times magnification.

**Figure 4:**
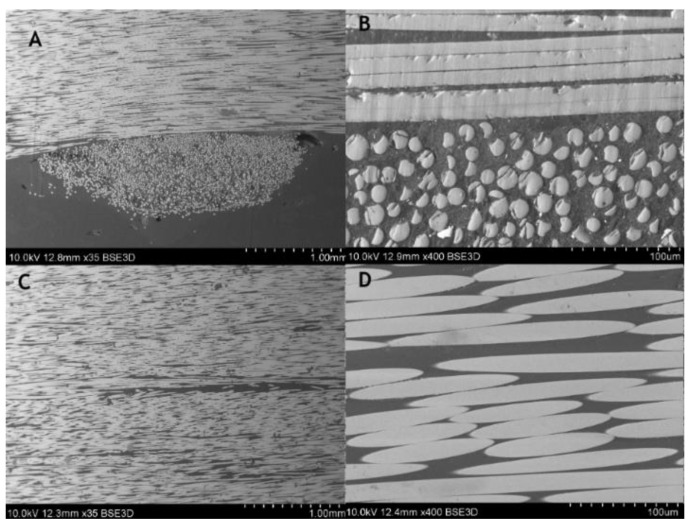
SEM images of composite rebar’s longitudinal section using (**A**) 35 times magnification, (**B**) 400 times magnification, outer part (**C**) 35 times magnification, (**D**) 400 times magnification, central part.

**Figure 5:**
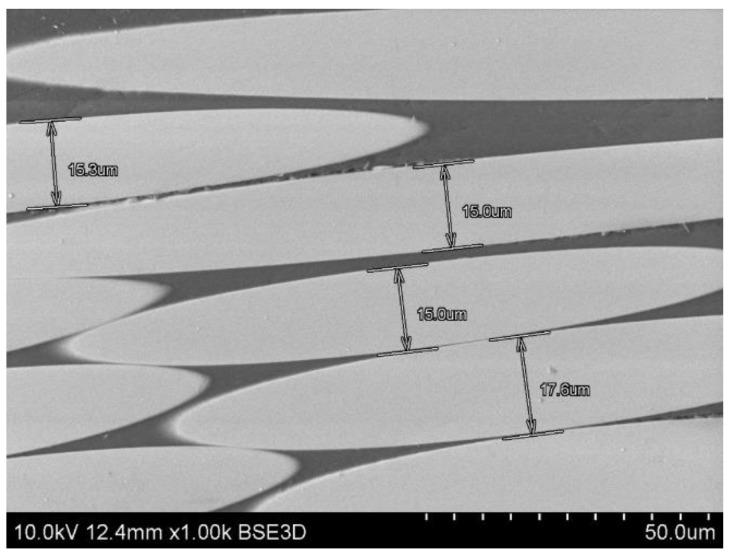
Measurement of the glass fiber diameters from a longitudinal cross-section of GFRP rebars.

For the composite material, quality evaluation is important to identify the microstructural features of composites like fiber distribution, evidence of voids and bubbles, fiber aggregation rate, as well as the measurement of the fibers diameters and its variability. Figure 3 shows the part of the rebar’s cross-section, and Figure 4 gives the longitudinal section of the specimen. In the observed microstructure it can be stated the uniformity of fibers’ distribution is relatively high, and the fibers are dominant without any inclusions. Comparable diameters of fibers warrant a good quality of fibers used by manufacturers. For the longitudinal cut sample, the SEM image shows 2 different parts, the outer (A, B), and the central (C, D) part of the rebar (Figure 4). Part A and B allow us to observe an overwrapped layer on the GFRP rod, in which fibers were cut transversally, and longitudinally in the major part of the rod. Other parts (C, D) show the distribution of the fibers in the central part of the rod. Also based on the measurements from Figure 5 it can be concluded that the glass fiber diameters vary from 15–17 µm.

**Figure 6:**
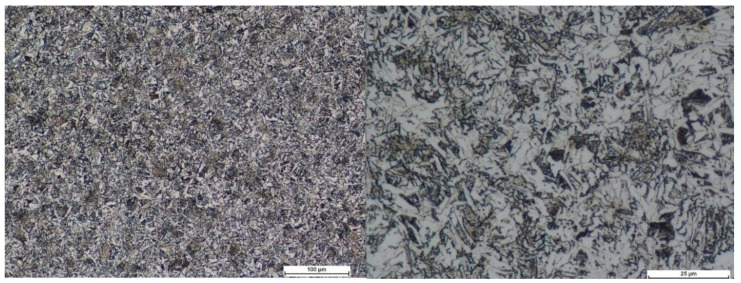
SEM images of steel B500SP rebars, typical microstructure from the outer part of the rebar (hardened).

For comparative analysis, we selected B500SP steel. The number 500 in the steel designation indicates the value of the characteristic yield strength in MPa, and SP marks its weldability. This steel meets, among others, the requirements of the PN-B-03264:2002 standard and is classified as the highest strength class A-IIIN. Also, for steel rebars, the homogeneous steel structure typical for the B500SP type of heat-treated steel (outer surface) was exhibited after etching 3%HNO_3_, see Figure 6.

### 2.2. Bending Test

This research aimed to compare the flexural strength of composite (ANKRA LLC) and steel (B500SP) rebars. The calculations of flexural strength factors and Young modulus for both rods were carried out for comparison in accordance with the ASTM D790 [34] and ASTM D7957 [17]. Considering that three-point bending can cause damage to the composite material at the point of load application, it was decided to use four-point bending. In four-point bending, there are lower contact stresses at the point of load and thus constant bending moment without shear forces at the pure bending section, which will allow to verify the usefulness of both methods in the analysis of composite rods. Regarding the mentioned standards, the four-point bending, and three-point bending tests were conducted using the universal testing machine MTS 810 (MTS Systems, Corporation, Eden Prairie, MN, USA). This device is controlled by a special software MTS Flex Test Console (Series 793, MTS Systems, Corporation, Eden Prairie, MN, USA). The working range of the hydraulic pulsator is equally ±100 kN with an additional load cell 5 kN. The specimen length in this test is equally 200 mm. For strain/deflection measurements, LVDT and MTS displacement gauges (clip-on gage with base 2.5 mm (+3/−1) mm travel arms—for elastic range) were used for registration of the deflection in load application points. Also, displacement signals were registered from the MTS 810 displacement transducer (cross head) of the tensile machine.

### 2.3. Radial Compression Test

**Figure 7:**
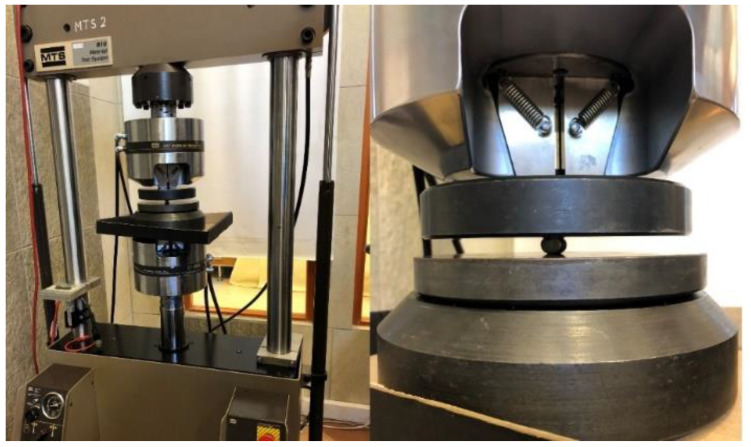
Specimen during the diametral compression test.

This test was focused on the longitudinal tensile strength of FRP rebars. The length is a crucial parameter that influenced the value of stress, so the Weibull distribution was applied to normalize the obtained results. During the diametral compression test, the composite rebar was loaded perpendicular to its long axis. Also, in this experiment, the MTS 810 testing machine was equipped with compression platen as is shown in Figure 7. The preparation of the samples included their cut for a specific length. In this research, three various lengths (10 mm, 20 mm, and 30 mm) were investigated with three repetitions.

### 2.4. Flexural Fatigue Test

**Figure 8:**
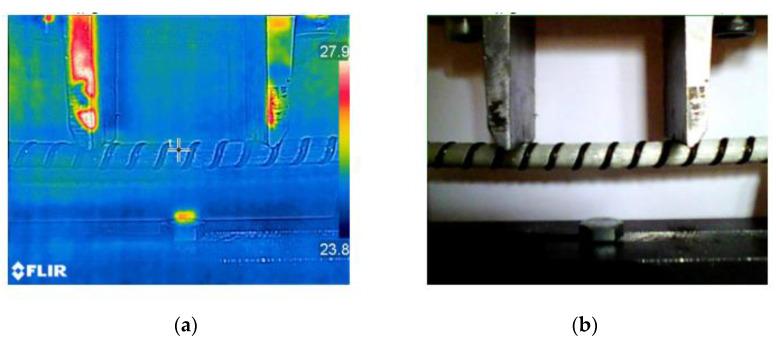
(**a**) Exemplary thermogram during cyclic bending test; (**b**) Specimen during the test.

The materials subject to static loads show greater strength than materials subjected to cyclic loads. This phenomenon is known as fatigue, and it is measurable by mechanical tests, which include repeating various stresses altering in a regular cycle from minimum to maximum value. Most fatigue testing machines use eccentric rotational weight for generating cyclic altering loads. To conduct this experiment, MTS 810 equipped with support for four-point bending was used. It accomplished the array measurement for the various value of the maximum load. The load used for the maximum load of composite rebars includes from 20–60% of force drives to failure of composite rebar obtain from bending tests. The frequency oscillates in the range of 1–1.5 Hz. In practical terms, fatigue testing of polymers, including composites, provides some difficulties, mainly related to the influence of frequency and thus generated heat. In the case of steel, the 1–10 Hz HCF (High Cycle Fatigue) range does not bring any changes. In the case of composites, thermal stability is no longer the same. Thus, the experimental observations with the use of a thermographic camera consisted of checking whether there is a significant difference in temperature during fatigue tests due to cyclic loading. In the presented experimental campaign, it was established that for low frequency *f*, no changes greater than 1–2 degrees C over a wide range of fatigue lifetimes were observed. For this purpose, the thermographic camera FLIR E6-XT (Wilsonville, OR, USA) was used. The exemplary thermogram obtained during cyclic loading is shown in Figure 8.

## 3. Results

### 3.1. Bending Results

**Figure 9:**
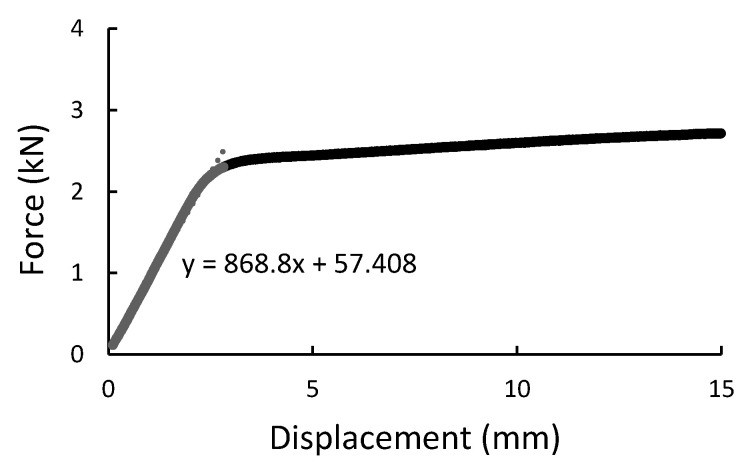
Three-point bending result presented as the force-displacement curve for B500SP steel rod.

**Figure 10:**
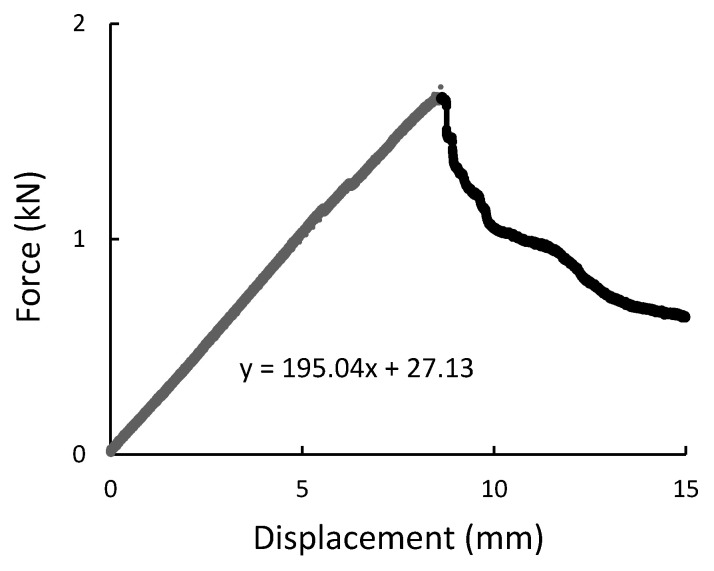
Three-point bending result presented as the force-displacement curve for composite rod.

**Figure 11:**
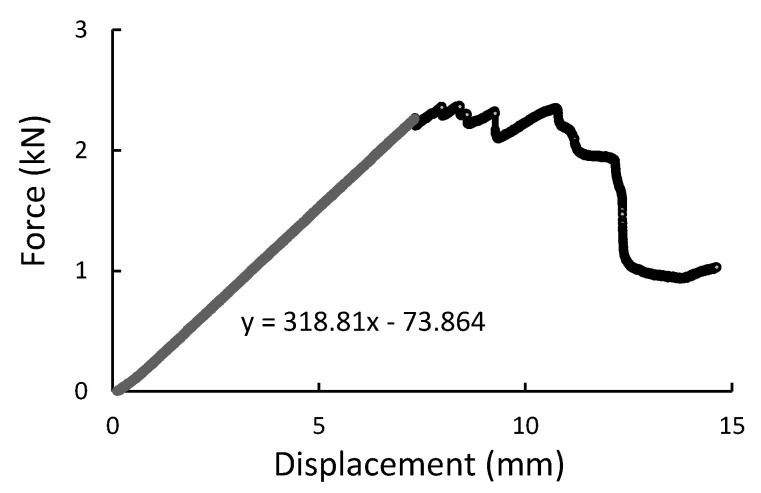
Four-point bending result presented as the force-displacement curve for composite rod.

**Figure 12:**
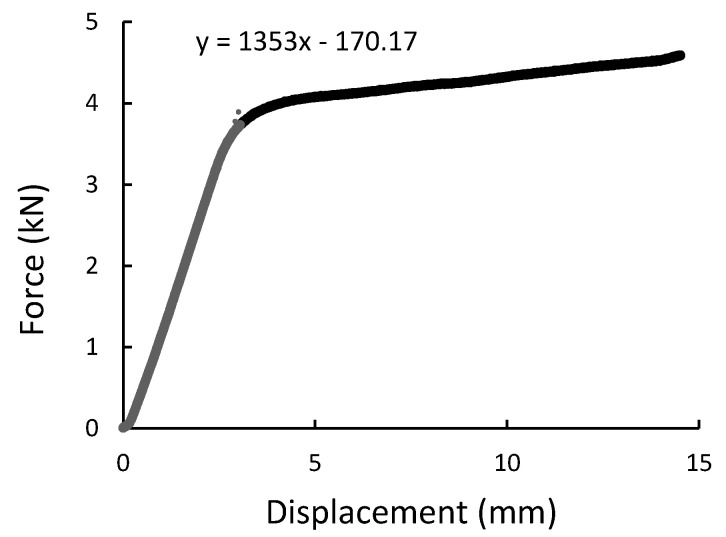
Four-point bending result presented as a force-displacement curve for B500SP steel rod.

During the experiment, the force, time, and displacement signals were registered. Representative force-displacement curves were plotted in Figure 9, Figure 10, Figure 11 and Figure 12. According to this date, the graphs were compiled for three-point bending (Figure 9 and Figure 10), and four-point-bending (Figure 11 and Figure 12) for steel, and composite rebar.

All results from three-point bending and four-point bending tests are summarized in Table 4.

In both 3PB and 4PB loading cases, the ductile behavior of B500SP material with a distinct plastic deformation and modulus of elasticity typical for structural steel is noted. The GFRP bar, on the other hand, has a higher brittleness with a local maximum force and a lower (about 3 times the modulus of elasticity). However, the final bending strength value results in higher results which is a good parameter in the application context.

### 3.2. Diametral Compression Test

**Figure 13:**
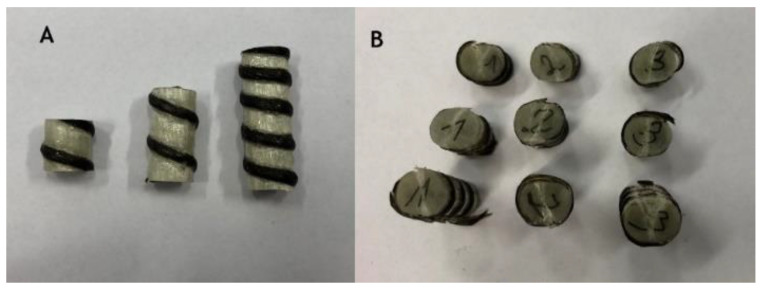
Samples used in diametral compression. (**A**) Before the test; (**B**) After compression test.

**Figure 14:**
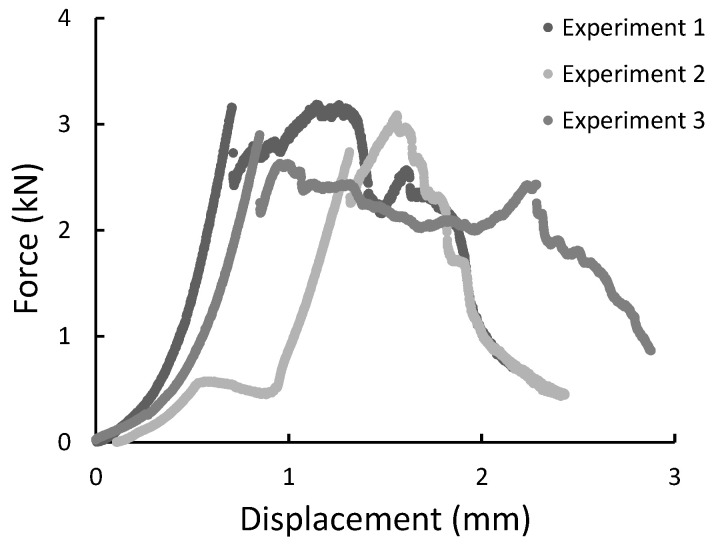
The force-displacement graph for composite specimens of 10 mm length registered during the diametral compression test.

**Figure 15:**
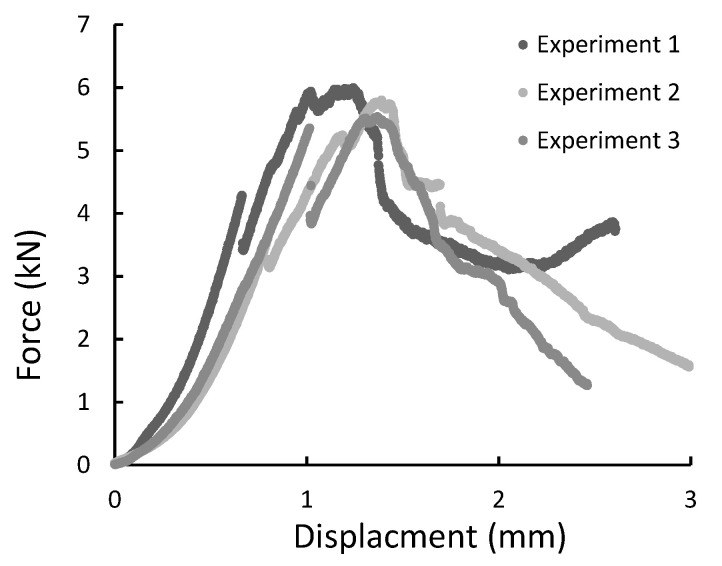
The force-displacement graph for composite specimens of 20 mm length registered during the diametral compression test.

**Figure 16:**
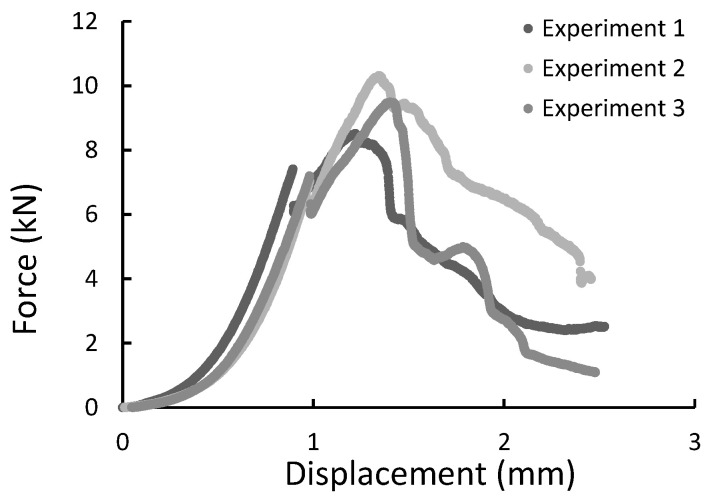
The force-displacement graph for composite specimens of 30 mm length registered during the diametral compression test.

All specimens during the diametral compression test failed in the central part of the rebar, fragmenting into 2 or 3 parts. Also, a flattening of the sample is noticeable, which did not return to the original shape. Figure 13 presents specimens used in the test before and after it. During the experiment, force-displacement curves were plotted and collected in Figure 14, Figure 15 and Figure 16.

While the circular rod is subjected to diametral compression, the central zone of the cylinder is subjected to biaxial stress. The normal stress in the perpendicular direction to applied load is tension stress, while in the parallel direction to the applied force is compression stress. The tension $\sigma_{x}$ and compression $\sigma_{y}$ stress in the central zone of the rod can be calculated using Equations (1) and (2) [35].

(1) $$\sigma_{x}=\frac{F}{\pi \cdot \frac{D}{2}\cdot L}\left(1-\frac{4\cdot {\left(\frac{D}{2}\right)}^{2}\cdot x^{2}}{{\left(R^{2}+x^{2}\right)}^{2}}\right)$$

(2) $$\sigma_{y}=\frac{F}{\pi \cdot \frac{D}{2}\cdot L}\left(1-\frac{4\cdot {\left(\frac{D}{2}\right)}^{2}}{{\left(R^{2}+x^{2}\right)}^{2}}\right)$$

where:

$\sigma_{x}$—diametral compression strength, 

*F*—maximum force,

*D*—diameter of the rod,

*L*—length of the rod.

*x*—distance from damage surface.

The analysis of Equation (1) shows that the maximum stress appears perpendicular to the failure surface (*x* = 0). The virtual transverse failure stress during elongation represents the load at break was calculated using Equation (1) assuming *x* = 0. The results obtained from the calculations are shown in Table 5.

**Figure 17:**
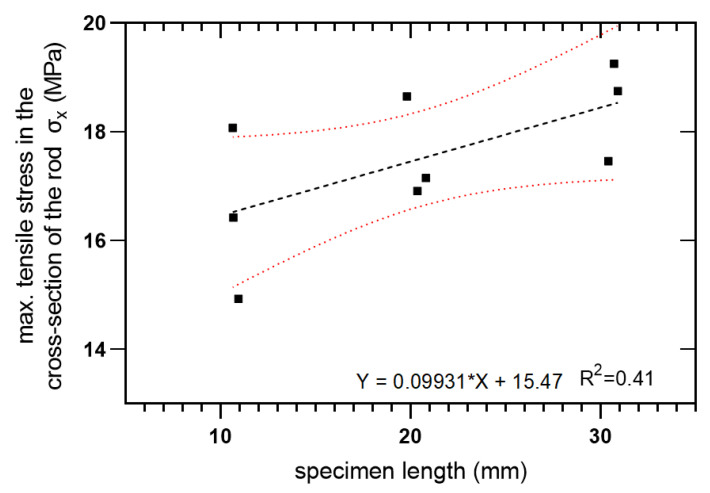
Transverse tensile strength as a function of specimen length.

The transverse tensile strength against specimen length is plotted in Figure 17 using linear regression and a 95% confidence level.

According to the Weibull distribution, the probability *F* that the rod of *L* length undergoes failure influenced by stress σ is described by Equation (3):

(3) $$F\left(\sigma \right)=1-\exp\left[-L{\left(\frac{\sigma }{\sigma_{0}}\right)}^{\alpha }\right],$$

where $\sigma_{0}$ and α are called scale and shape parameters. These two parameters may be determined by estimation of the highest probability. Especially, the parameters α and $\sigma_{0}$ are connected with strength data, according to Equations (4) and (5):

(4) $$\alpha =\frac{n}{\sigma_{0}^{-\alpha }(\sum_{i=1}^{n}L_{i}\sigma_{i}^{\alpha }ln\sigma_{i})-\sum_{i-1}^{n}ln\sigma_{i}},$$

(5) $$\sigma_{0}={\left[\frac{1}{n}\sum_{i=1}^{n}L_{i}\sigma_{i}^{\alpha }\right]}^{\frac{1}{\alpha }},$$

For the investigated composite material, the scale parameter equals $\sigma_{0}=14.262$ and the shape parameter α=15.715. The normalized strength was calculated using Equation (6):

(6) $$\bar{\sigma }=\frac{\sigma }{\sigma_{0}L^{\frac{-1}{\alpha }}},$$

Transverse tensile strength was calculated by Equation (7):

(7) $$\sigma_{med}=\sigma_{0}{\left(\frac{-ln0.5}{L}\right)}^{\frac{1}{\alpha }},$$

According to these formulas, Table 6 was composed. It shows the values of stress σ, normalized strength $\bar{\sigma }$, and the probability of failure *F(σ)*. The observed length effect on the probability of failure is noticeable–in Table 6 with increasing *L*, the probability of failure increases too. This seems to be consistent with the Weibull theory.

### 3.3. Fatigue Bending Test Results

During the fatigue test, the specimens were loaded by cyclic force *ΔF*, in which maximum values ranged from 20 to 60% of the critical bending stress calculated from the bending test 3PB/4PB. The experiment was conducted with a frequency of 1–1.5 Hz keeping a constant stress ratio *R* = *σ_min_/σ_max_* = 0.1. During the experiment, the number of cycles to failure (criterion–stiffness drop at 40%) was registered. For fatigue characterization, the S-N curves were drawn. The object of interest was the high cycle fatigue regime–which can be described by the power-law relationship:

(8) $$\sigma_{max}=A{\left(N_{f}\right)}^{n},$$

where: *A*, *n*-experimentally determined parameters in HCF regime (high cycle fatigue).

**Figure 18:**
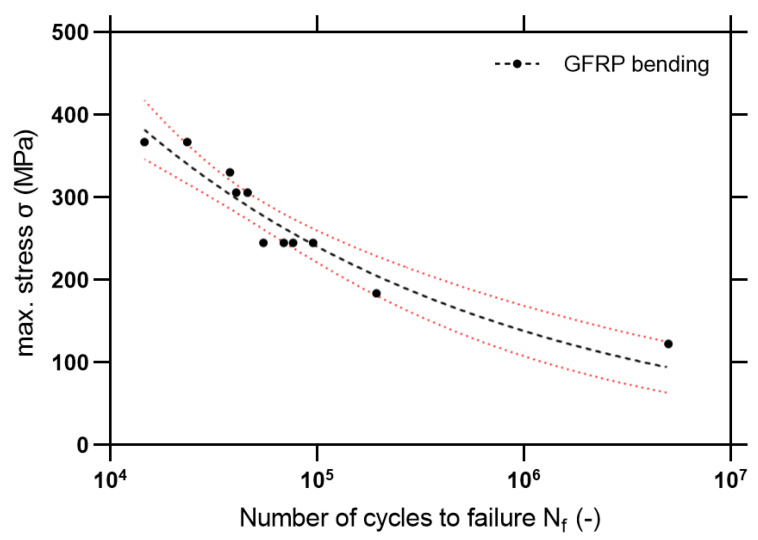
S-N curve for composite rebars with marked 95% confidence level.

According to the obtained results, the S-N curve for composite rebar was compiled (Figure 18). The slope parameter *m* was calculated as:

(9) $$m=\frac{-1}{n}.$$

**Figure 19:**
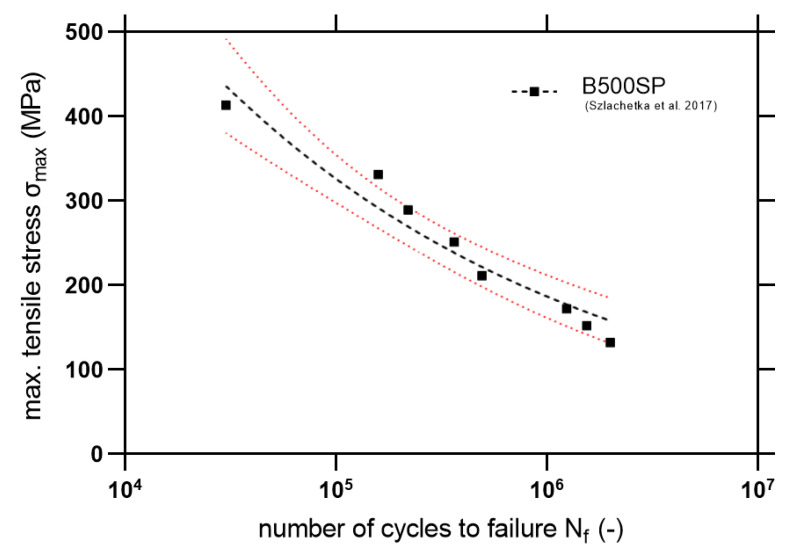
S-N curve for B500SP steel rebars with marked 95% confidence level (fatigue tensile test).

For the comparison of the fatigue properties, Szlachetka et al. [36] discussed the fatigue phenomenon of the ribbed steel rebars under tensile mode fatigue (using *R* = 0). Based on the data available in [36] the S-N curve with 95% confidence intervals is plotted in Figure 19. Also, statistical outputs are computed in Table 7.

Based on Equations (8) and (9), statistical outputs are calculated in Table 7. It is noticeable that in a high cycle fatigue regime, there is a higher slope of the S-N curve for the steel material. Also noticeable is the slightly better fatigue resistance for higher stress levels, which is confirmed by the better ductility of the material. However, for fatigue lifetimes close to 10^6^ cycles, GFRP exhibit similar fatigue lifetimes in comparison with steel. However, it is important to be particularly careful in drawing final comparative conclusions since both materials were tested with different conditions—the steel in tension and the composite in bending. However, the observed fatigue properties encourage further studies on the programmable structure of the composites—in pultrusion, it is possible to shape the geometry and cross-section in any way to ensure optimal fiber arrangement. Fatigue damage analysis using SEM will be an important part of the study.

**Figure 20:**
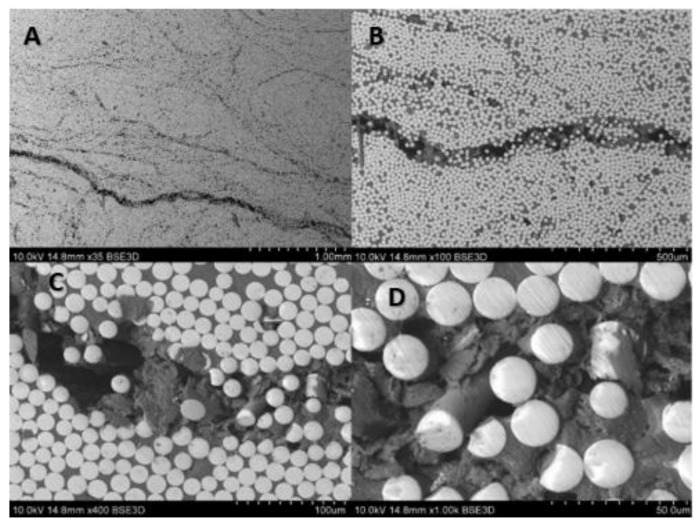
SEM images after fatigue test (max. load 1500 N) of composite rebar’s cross-section using (**A**) 35 times magnification, (**B**) 100 times magnification, (**C**) 400 times magnification, (**D**) 1000 times magnification.

**Figure 21:**
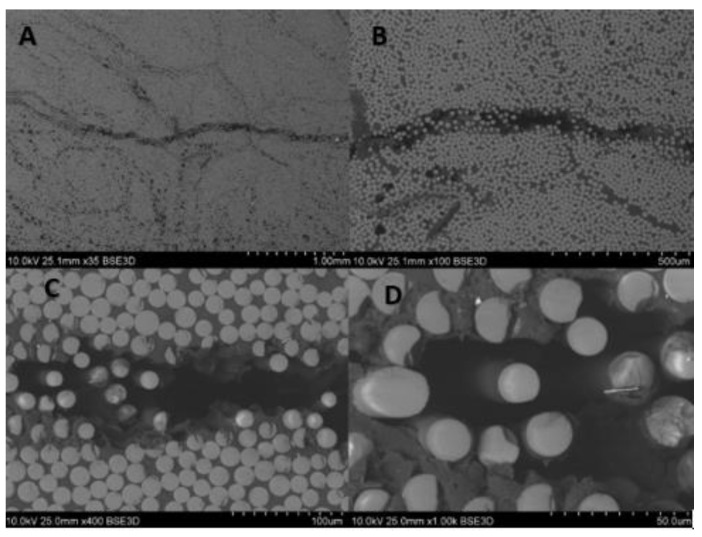
SEM images after fatigue test (max. load 1000 N) of composite rebar’s cross-section using (**A**) 35 times magnification, (**B**) 100 times magnification, (**C**) 400 times magnification, (**D**) 1000 times magnification.

**Figure 22:**
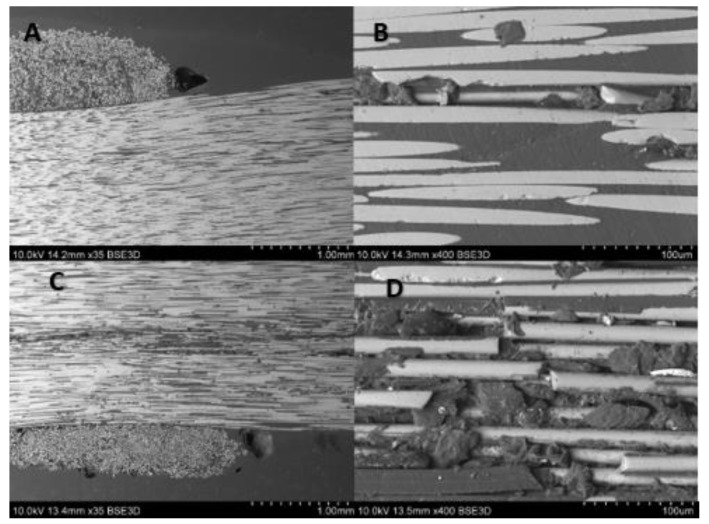
SEM images after fatigue test (max. load 1500 N) of composite rebar’s longitudinal section using (**A**) 35 times magnification of tensile part, (**B**) 400 times magnification of tensile part, (**C**) 35 times magnification of compressed part, (**D**) 400 times magnification of compressed part.

**Figure 23:**
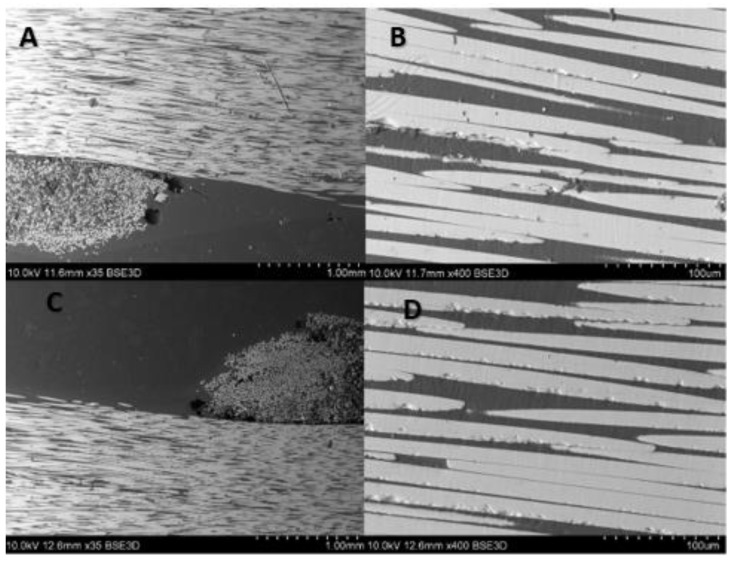
SEM images after fatigue test (max. load 1000 N) of composite rebar’s longitudinal section using (**A**) 35 times magnification of tensile part, (**B**) 400 times magnification of tensile part, (**C**) 35 times magnification of compressed part, (**D**) 400 times magnification of compressed part.

The fatigue fracture of B500SP material is a typical and well-known fatigue damaged–as it is evidenced in [36]. Due to the higher interest in the preliminary fatigue study of GFRP rebars, it was extremely important to perform, after the fatigue tests, the microscopic analysis of damaged specimens. The selected samples were prepared for SEM (HITACHI S-3400N) investigations. The two samples were selected for examination of the damage mechanism of the composite rebars. The first one was subjected to the force of 1500 N, with frequency 1 Hz withstood 14 649 cycles. The force equal 1000 N, and frequency 1 Hz acted on the second sample withstood 76 636 cycles. Once more, the transverse, and longitudinal microsection of investigated rebars were taken. Figure 20 and Figure 21 show the cross-section of the rebars after the experiment. Further, Figure 22 and Figure 23 present the longitudinal microsection.

Cross-sectional images (Figure 20 and Figure 21) show noticeable delaminations and fiber fracture in both specimens, however, it is more severe for the specimen subjected to the greater maximum load (Figure 20). The larger force likely drives this, inducing higher stress in fibers. The longitudinal microsections (Figure 22 and Figure 23) give information about the zones where the fibers were elongated (A, B), and compressed (C, D). In the longitudinal section of both specimens, the fiber fracture can be observed even with the lowest magnitude, most evidently in the compressed part of the specimens. It is much more prominent in the specimen subjected to the greater maximum load (Figure 22). This kind of damage may be ascribed to fiber microbuckling, which is the typical behavior of reinforcing fibers after exceeding the critical compressive load (fibers behaving like slender columns embedded in a relatively soft matrix). This fiber buckling subsequently causes delamination.

## 4. Conclusions

Based on the experimental research, the following conclusions can be drawn:

- The elastic modulus of GFRP rebars is significantly lower than for steel rebars (approx. 3x). It is worth underlining the higher static flexural properties (similar for 3PB and 4PB test) of GFRP materials than high-quality B500SP steel (see Table 4).
- Looking at the specimens with a higher scale (*σ*_0_) loaded with a higher stress level, the failure processes such as delamination and fiber fracture can be observed. The higher stress level in fibers is influenced by the greater force, driving this phenomenon. In the longitudinal sections of investigated samples, even the lowest magnitude provides the locations of the fractures. The inferred failure mode is fiber buckling under compressive load exceeding the critical load for slender fibers.
- The results obtained from fatigue tests (*m* = 4.33, *A* = 4692 MPa) encourage further analysis. Considering previous conclusions, the diametral compression test reflects the weaker nature of the composite materials based on the interlaminar compressive strength. The proposed methodology allows us to describe invariantly the experimental transversal strength of the composite materials. For tested GFRP rebars (epoxy resin) *σ_med_* varies from 17.38–18.60 MPa independent of the scale length.
- Considering the expected durability of the structure, the failure mechanism is likely to significantly improve their fatigue behavior under the influence of cyclic bending. It was expected to obtain greater resistance in extreme fibers as a result of single line wrapping. It seems that the reasonable direction of searching for reinforcements of composite structures should be the improvement of the bearing capacity of outer layers.
- In comparison with steel rebars (fatigue tensile test), the obtained results for GFRP are comparable in the HCF regime. It is worth noting that in near fatigue endurance regime (2–5 × 10^6^ cycles) both rebars exhibit similar behavior.
- Further studies considering the environmental influence on fatigue endurance both GFRP and steel rods are necessary regarding the possible anti-fatigue design of the geometry of the rebars using pultrusion process in order to redistribute damage stress in the outer part of the composite rods.

## Figures and Tables

**Table 1:** Basic properties of rebars’ reinforcement.

Properties	Reinforcement				
	Steel	CFRP	AFRP	GFRP	BFRP
Longitudinal coefficient of thermal expansion (×10^−6^/°C)	11.7	(−9.0)–0.0	(−6.0)–(−2.0)	6.0–10.0	n/a
Transverse coefficient of thermal expansion (×10^−6^/°C)	11.7	74.0–104.0	60.0–80.0	21.0–23.0	n/a
Density (g/cm^3^)	7.86	1.50–1.60	1.25–1.40	1.25–2.10	1.90

CFRP–Carbon fiber reinforced polymer, AFRP–Aramid fiber reinforced polymer, GFRP–Glass fiber reinforced polymer, BFRP–Basalt fiber reinforced polymer.

**Table 2:** Mechanical properties of Fiber Reinforced Polymer (FRP) rebars.

Properties	Reinforcement				
	Steel	CFRP	AFRP	GFRP	BFRP
YS (MPa)	276–517	-	-	-	-
UTS (MPa)	483–690	600–3690	1720–2540	483–1600	1100
Young Modulus (GPa)	200	120–580	41–125	35–51	70
Elongation at break (%)	6.0–12.0	0.5–1.7	1.9–4.4	1.2–3.1	2.2

YS—Yield strength, UTS—Ultimate tensile strength.

**Table 3:** Tested rebar properties made from glass fibers.

Property	R_m_ (MPa)	A_5_ (%)	ρ (kg/m^3^)
**Value**	1000	2.5–5	1900

R_m_—Ultimate tensile strength, A_5_—Elongation at fracture, ρ—density.

**Table 4:** Results from the three and four-point bending test.

	Three-Point Bending (3PB)		Four-Point Bending (4PB)
	Young Modulus (GPa)	Flexural Strength (MPa)	Flexural Strength (MPa)
**Steel rebar**	218.4	706.9	614.2
**GFRP Composite rebar**	76.2	999.3	973.4

**Table 5:** Summarized results of the diametral compression test.

Lp.	L (mm)	F (N)	*σ_x_* (MPa)
1	10.64	3085	18.07
2	10.68	2814	16.42
3	10.95	2624	14.93
4	19.8	5926	18.65
5	20.8	5724	17.15
6	20.35	5520	16.91
7	30.4	8515	17.46
8	30.9	9298	18.75
9	30.71	9485	19.25

**Table 6:** Calculated values from the diametral compression test.

L (mm)	F (N)	σ_0_ (MPa)	$\bar{\sigma }$	F (σ) (-)	$\sigma_{med}\;\left(MPa\right)$
10.64	3085.0	18.07	1.09	0.36	18.60
10.68	2814.0	16.42	0.99	0.09	18.60
10.95	2624.0	14.93	0.90	0.02	18.57
19.80	5926.0	18.65	1.08	0.74	17.88
20.80	5724.0	17.15	0.99	0.31	17.83
20.35	5520.0	16.91	0.98	0.26	17.85
30.40	8515.0	17.46	0.98	0.52	17.40
30.90	9298.0	18.75	1.06	0.90	17.38
30.71	9485.0	19.25	1.09	0.97	17.39

**Table 7:** Statistical outputs of the power-law data fitting for HCF (high cycle fatigue regime > 10^4^ cycles).

Material	*A*	*N*	m	*R* ^2^
GFRP	4692.0	−0.231	4.33	0.94
B500SP	5398.0	−0.273	3.66	0.89

## Data Availability

The data presented in this study are available on request from the corresponding author.
